# IWR-1 inhibits epithelial-mesenchymal transition of colorectal cancer cells through suppressing Wnt/β-catenin signaling as well as survivin expression

**DOI:** 10.18632/oncotarget.4354

**Published:** 2015-09-16

**Authors:** Sang Chul Lee, Ok-Hee Kim, Sang Kuon Lee, Say-June Kim

**Affiliations:** ^1^ Department of Surgery, Daejeon St. Mary’s Hospital, College of Medicine, The Catholic University of Korea, Daejeon, Republic of Korea

**Keywords:** IWR-1, survivin, epithelial mesenchymal transition (EMT), colorectal cancer, wnt/β-catenin signaling

## Abstract

Aberrant activation of Wnt/β-catenin signaling is frequently observed in patients with colorectal cancer (CRC) and is considered a major determinant of CRC pathogenesis. CRC pathogenesis is particularly accompanied by epithelial-mesenchymal transition (EMT) and survivin expression. Here, we investigated the potential and mechanism of a novel Wnt/β-catenin inhibitor IWR-1 to suppress tumor metastasis in relation with EMT and survivin expression. We first determined the EMT reversal effects of IWR-1 in *in vitro* (HCT116 and HT29 cells) and *ex vivo* (specimens of CRC patients) CRC models. It was shown that IWR-1 inhibited cell proliferation and EMT even in the presence of TNF-α-induced cancer cell stimulation. IWR-1 also significantly suppressed cell migration, invasion, and matrix metalloproteinase activities of CRC cell lines. Furthermore, we showed the evidence that IWR-1 provides EMT reversal effects by directly suppressing survivin expression by the followings: 1) IWR-1 could not completely inhibit EMT in survivin-overexpressing HCT116 cells, 2) EMT reversal effects of IWR-1 were more pronounced in survivin-suppressed cells, and 3) Survivin promoter assay directly identified the survivin promoter region responsible for inhibition of survivin transcription by IWR-1. Taken altogether, our results demonstrate that IWR-1 has the potential to suppress tumor metastasis by inhibiting Wnt/β-catenin pathway as well as survivin expression. Therefore, IWR-1 could be considered for future clinical use as a therapeutic agent to treat CRC.

## INTRODUCTION

Colorectal cancer (CRC) is diagnosed in more than 1 million people worldwide each year, and is responsible for the death of approximately half of these patients, making it the fourth leading cause of cancer death in the world [1]. Wnt/ β-catenin pathway serves critical functions in CRC oncogenesis, especially for inducing proliferation and differentiation in 90% CRCs [2, 3]. Without Wnt stimulation, β-catenin is incorporated in the β-catenin destruction complex with APC, Axin, and glycogen synthase kinase 3β (GSK3β), and is to be phosphorylated and processed to undergo proteasomal degradation. However, when Wnt ligands bind Frizzled receptors, Disheveled proteins are activated, and then β-catenin degradation is blocked. Subsequently, excess β-catenin enters the nucleus where it orchestrates the transcription of several target genes responsible for cell proliferation [4].

The Wnt/β-catenin pathway is the one of the key elements inducing epithelial-mesenchymal transition (EMT) [5, 6]. During EMT, epithelial cells acquire the characteristics of mesenchymal cells, such as lack of polarization, increased motility and invasiveness, decreased cell-cell junctions, and chemotherapeutic resistance [7, 8]. Thus, EMT is known to be essential for the initial and overall rate-limiting steps of CRC invasion and metastasis [7]. Furthermore, EMT is also considered a major determinant of CRC prognosis because the deep invasion and distal metastasis considerably worsen the prognosis of CRCs [9]. In recent years, as the emphasis of EMT in the tumor progression has been increased, anticancer strategies against EMT have been widely investigated [10–12].

Survivin (BIRC5), a member of the inhibitor of apoptosis (IAP) protein family, is a representative anti-apoptotic protein which promotes tumor cell growth [13]. Survivin is abundantly expressed during fetal development in humans, but is rarely present in adult tissues [14]; however, most human cancer cells express survivin, including CRC cells which were reported to express survivin up to 68% [15]. It has been reported that survivin expression correlates with advanced disease, poorer survival, and chemotherapy and radiation resistance [14, 16–18]. Therefore, survivin is of increasing interest as a potential therapeutic target to inhibit cancer growth [19, 20]

Evidence has shown that the Wnt/β-catenin pathway one of the key factors inducing survivin expression [14, 21, 22]. Despite the significance of the Wnt/β-catenin pathway in CRC, anticancer agents targeting Wnt/β-catenin signaling have not been extensively investigated. IWR-1 was first identified in 2009 as one of the inhibitors of Wnt response (IWR) [23]. It has been shown that IWR-1 promotes β-catenin destruction in the β-catenin destruction complex by abrogating Axin protein turnover and thereby inducing β-catenin phosphorylation, a prerequisite for proteasome-mediated β-catenin degradation [23]. However, the roles of IWR-1 in the CRC pathogenesis, especially in relation with EMT, are still not fully understood and thus need to be clarified. The aim of our work was to elucidate the role of IWR-1 in CRC dissemination, especially in relation with EMT and survivin expression and to provide insights into the potential and mechanism of IWR-1 to suppress CRC invasion and metastasis.

## RESULTS

### IWR-1 effects on the HCT116 cell proliferation and EMT

**Figure 1:**
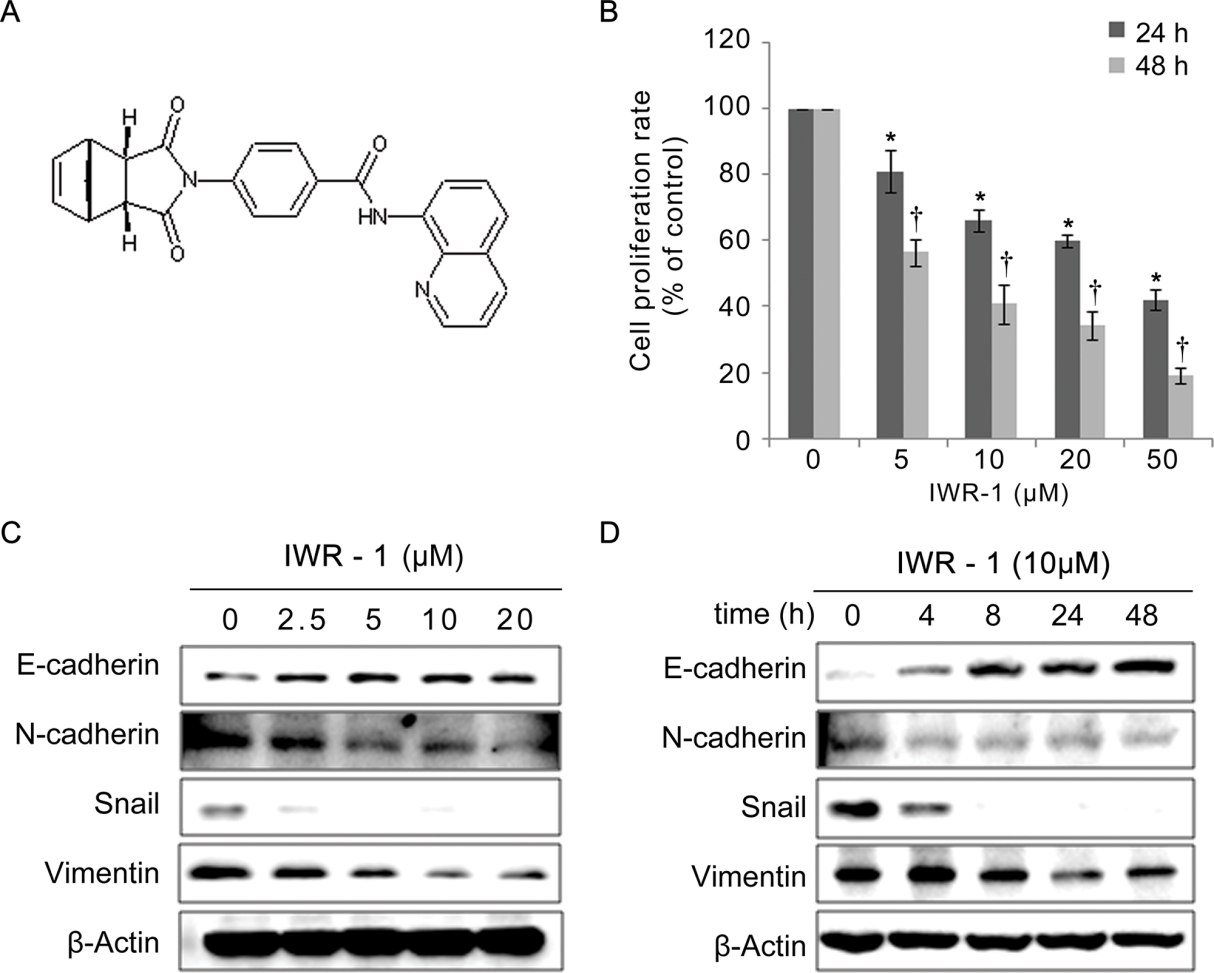
Effects of IWR-1 on proliferation and EMT in HCT116 colon cancer cells **A.** Chemical structure of IWR-1. **B.** Treatment of HCT116 cells with increasing concentrations of IWR-1 for 24 h and 48 h. IWR-1 decreased the proliferation of HCT116 cells in a dose- and time-dependent manner. **C, D.** Western blot analyses showing protein expression patterns of the EMT markers according to IWR-1 dose and exposure time. β-Actin was used as a loading control. Values represent means ± SD of three independent experiments. *P < 0.05, † *P* < 0.05.

Human colorectal cancer HCT116 cells were treated with increasing concentrations (5–50 μM) of IWR-1 (structure is shown in Figure 1A) for 24 h or 48 h and analyzed for cell proliferation. The results indicate that IWR-1 decreased the proliferation of HCT116 cells in a dose- and time-dependent manner (Figure 1B).

The epithelial marker E-cadherin and the mesenchymal markers N-cadherin, Snail, and Vimentin are specifically used to monitor the EMT process. Expression analysis of these markers in IWR-1-treated HCT116 cells by western blotting revealed that IWR-1 dose- and time-dependently increased the levels of the epithelial marker E-cadherin, whereas it decreased the mesenchymal markers N-cadherin, Vimentin and Snail (Figure 1C–1D). Collectively, our results indicate that IWR-1 effectively inhibited the EMT process in HCT116 cells. Similar results were obtained from the HT29 cell line (see the Supplementary Figure S1A–S1B).

### IWR-1 effects in the TNF-α-induced EMT in HCT116 cells

**Figure 2:**
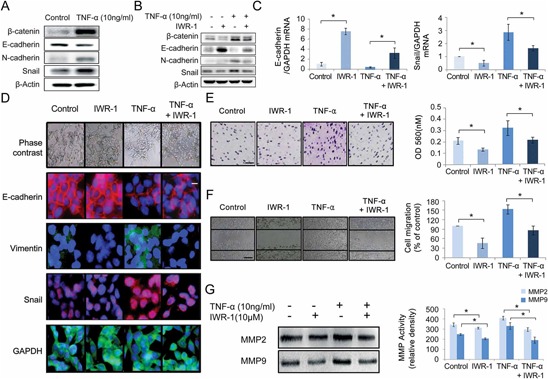
IWR-1 effect on TNF-α-induced EMT, cell invasion, migration and MMP activities in HCT116 cells **A.** Western blot analysis showing that TNF-α increased the expression of β-catenin, induced EMT-like expressional changes, and caused a switch from E-cadherin to N-cadherin expression in HCT116 cells. β-Actin was used as loading controls. **B.** Western blot analysis showing IWR-1 effects on the expressions of β-catenin and EMT markers. IWR-1 decreased the expression of β-catenin and inhibited EMT progression, even in the presence of TNF-α stimulation in HCT116 cells. **C.** RT-qPCR showing that the mRNA levels of E-cadherin and Snail were increased and decreased after IWR-1 treatment, respectively (*P* < 0.05). **D.** Immunofluorescence analysis demonstrating the increase in E-cadherin and decrease in Vimentin and Snail after the treatment of IWR-1, even in the TNF-α-stimulated HCT116 cells. GAPDH was used as loading controls. **E.** Transwell invasion assay (magnification, × 100, scale bar 20 μM) showing that IWR-1 significantly inhibited TNF-α-stimulated HCT116 cell invasion (*P* < 0.05). **F.** Wound-healing assay (magnification, × 200, scale bar 50 μM) showing that IWR-1 significantly inhibited TNF-α-stimulated HCT116 cell migration (*P* < 0.05). **G.** Quantitative gelatin zymography showing IWR-1 effects on the proteolytic MMP2 and MMP9 activities in both untreated and TNF-α-treated HCT116 cells. IWR-1 significantly reduced the activity of MMP2 and MMP9 in HCT116 cells regardless of TNF-α stimulation. Values represent the means ± SD of three independent experiments. *p < 0.05

It has been shown that TNF-α induces EMT in human HCT116 cells and thereby promotes CRC invasion and metastasis [24]. We were intended to investigate the EMT-suppressive effect of IWR-1 in the presence of EMT overstimulation. We thus analyzed the expression of the EMT markers and Wnt component β-catenin in HCT116 cells after stimulation with 10 ng/ml TNF-α for 24 h. As expected, TNF-α stimulation increased β-catenin expression, and induced EMT-like expressional changes, such as increased N-cadherin and Snail and decreased E-cadherin expressions (Figure 2A). IWR-1, however, decreased β-catenin expression, and inhibited the EMT-like expressional changes whereby decreasing N-cadherin and Snail and increasing E-cadherin expressions, even in the presence of TNF-α-induced EMT stimulation (Figure 2B). Subsequently, RT-qPCR demonstrated IWR-1 effect of inhibiting EMT(increasing E-cadherin and decreasing Snail) in the mRNA levels under the TNF-α-induced EMT stimulation (Figure 2C). These observations were substantiated by immunofluorescence microscopy, which also showed the increase of E-cadherin and decrease of Vimentin and Snail after the treatment of IWR-1, even in the presence of TNF-α-induced EMT stimulation (Figure 2D). Similar results were obtained from the HT29 cell line (see the Supplementary Figure S2A–S2D). We also have shown that IWR-1 equally inhibits EMT of the colon carcinoma cell lines with (HT29, SW480, and SW620 cells) or without (HCT116 cells) APC mutation (see the Supplementary Figure S3).

### IWR-1 effects on HCT116 cell migration and invasion

To determine the IWR-1 effects on CRC invasion, we conducted the Transwell invasion assay. Although TNF-α enhanced the invasiveness of HCT116 cells, IWR-1 significantly inhibited TNF-α-induced cell invasion (*P* < 0.05) (Figure 2E). Subsequently, to determine IWR-1 effects on CRC migration, we conducted the wound healing assay. Similar to cell invasion, although TNF-α enhanced HCT116 cell migration, IWR-1 significantly inhibited TNF-α-stimulated migration (*P* < 0.05) (Figure 2F). Similar results were obtained from the HT29 cell line (see the Supplementary Figure S4A–S4B). Taken together, we clearly demonstrated that IWR-1 significantly inhibited HCT116 and HT29 cell invasion and migration, even in the presence of TNF-α-induced cancer cell stimulation.

To further investigate the role of IWR-1 in CRC invasion, we measured matrix metalloproteinase (MMP) activities. MMPs, mostly MMP2 and MMP9, are known to facilitate cancer cell invasion by degrading extracellular matrix (ECM) proteins, and are thus increased during EMT [25, 26]. We determined MMP2 and MMP9 activities in HCT116 cells by gelatin zymography followed by densitometry analysis. It was shown that although TNF-α increased gelatin degradation by MMP2 and MMP9 in HCT116 cell supernatants, IWR-1significantly reduced the MMP2 and MMP9 activities in the cancer cells, even in the presence of TNF-α-induced cancer cell stimulation (Figure 2G).

### IWR-1 effects according to the Akt expression status

**Figure 3:**
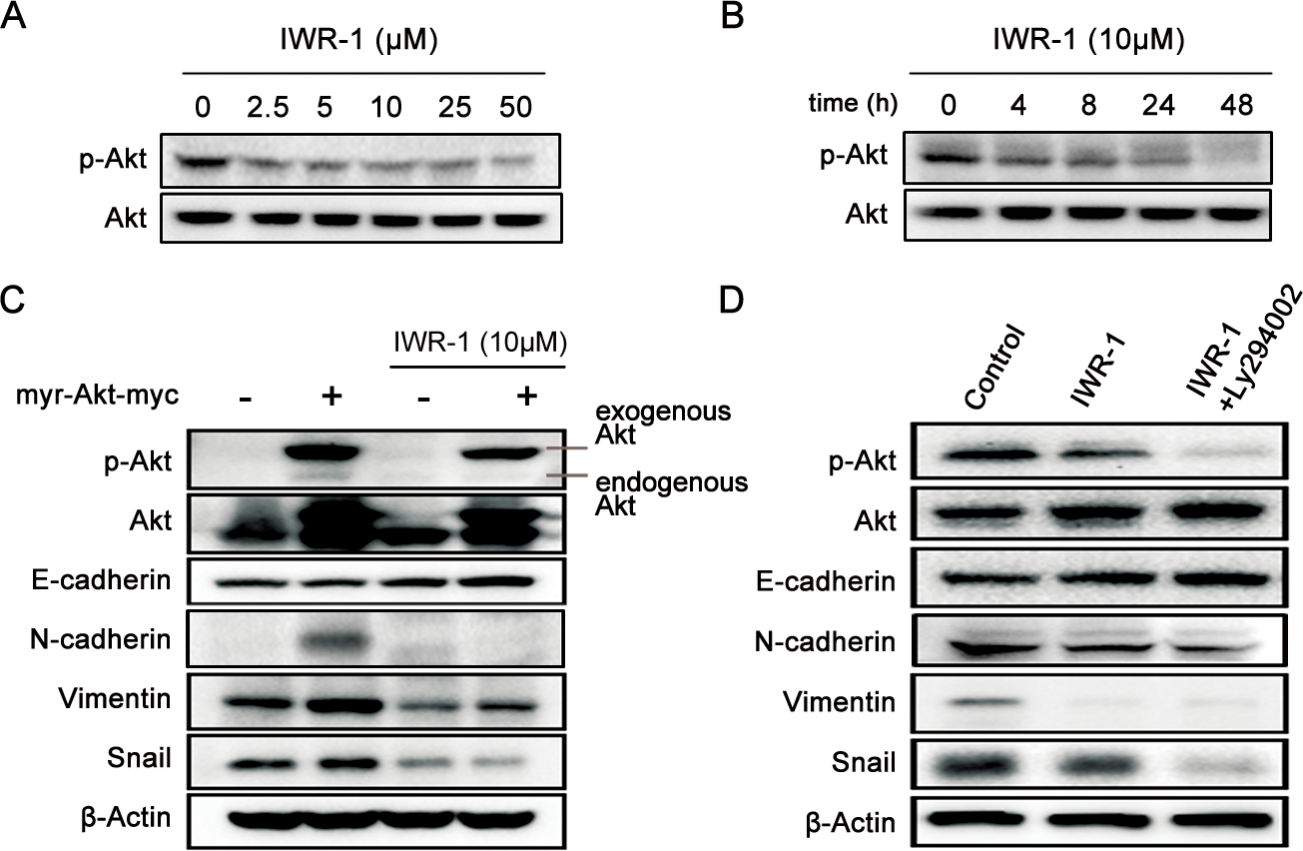
IWR-1 effects on EMT according to the Akt expression status **A–B.** Western blot analysis showing that IWR-1 decreased the phosphorylation of Akt in a concentration- and time-dependent manner, respectively. **C.** IWR-1 effects on the HCT116 cells exhibiting activated Akt constructs (transfected with pcDNA3.1Myr-Akt). EMT-suppressing effect of IWR-1 was also demonstrated following Akt overexpression. **D.** IWR-1 effects on the HCT116 cells with inhibited Akt (treated with 20 μM PI3K inhibitor LY294002). EMT-suppressing effect of IWR-1 was also demonstrated following Akt suppression.

Activation of PI3K/Akt signaling has been reported in 60–70% of human CRCs and PI3K/Akt inhibitors have been suggested as potential therapeutic agents [27]. We, therefore, investigated the involvement of Akt activation in IWR-1 inhibition of EMT in human CRC cells. It was shown that IWR-1 effectively inhibited Akt expression in a dose- and time-dependent manner (Figure 3A, 3B).

To determine the precise effect of IWR-1 according to the Akt activity, we measured the EMT-suppressing effect of IWR-1 after Akt overexpression or suppression. Akt overexpression and suppression were made by the transfection with pcDNA3.1Myr-Akt and by the treatment with 20 μM PI3K inhibitor LY294002, respectively. It was appeared that Akt effectively suppressed EMT processes regardless of whether Akt was overexpressed or suppressed (Figure 3C, 3D). These results suggest that IWR-1 does not directly affect Akt in suppressing EMT.

### IWR-1 effects on the survivin expression

**Figure 4:**
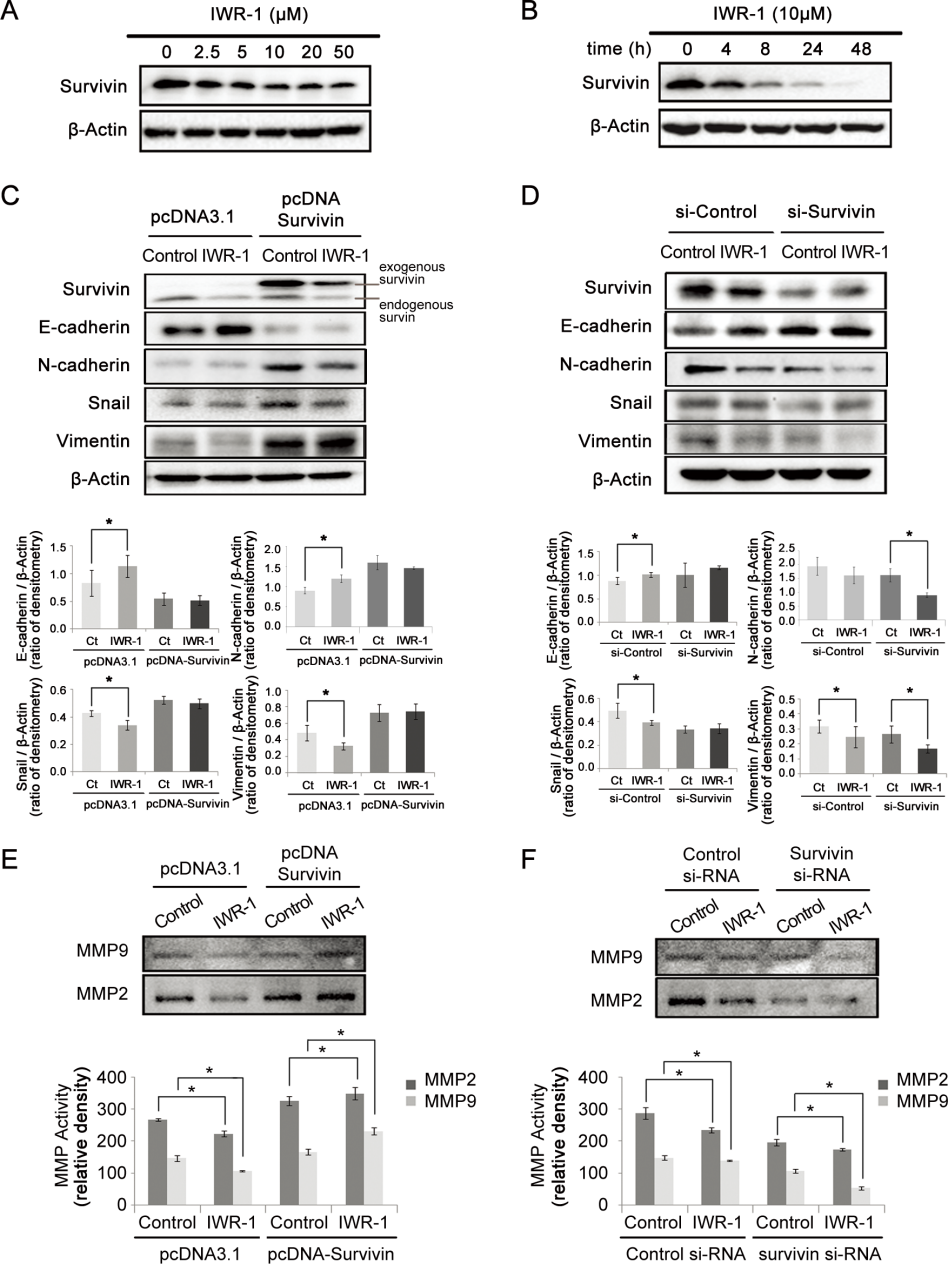
IWR-1 effects on EMT according to the survivin expression status **A–B.** Western blot analysis showing that IWR-1 decreased the survivin expression in a concentration- and time-dependent manner, respectively. **C.** IWR-1 effects in the survivin-overexpressing HCT116 cells which had been transfected with pcDNA-Survivin. The control group was transfected with empty pcDNA3.1 vector. Note that IWR-1 could not effectively inhibit EMT in survivin-overexpressing HCT116 cells. **D.** IWR-1 effects in the survivin-suppressed HCT116 cells which had been treated with si-RNA targeting survivin (si-Survivin). The control group was treated with scrambled siRNA (si-Control). EMT-suppressing effects of IWR-1 was more pronounced in survivin-suppressed HCT116 cells than in the control HCT116 cells. **E.** Gelatin zymography showing IWR-1 effects on the activities of MMP2 and MMP9 in the survivin-overexpressing HCT116 cells (pcDNA-Survivin). Note that IWR-1 could not effectively reduce MMP activities in survivin-overexpressing HCT116 cells. **F.** Gelatin zymography showing IWR-1 effects on the activities of MMP2 and MMP9 in the survivin-suppressed HCT116 cells (si-Survivin). Reducing MMP activities by IWR-1 was more pronounced in survivin-suppressed HCT116 cells than in the control HCT116 cells.

Next, we investigated IWR-1 effects on survivin. Survivin is known as one of the Wnt target gene [28] and a downstream of PI3K/Akt signaling as well [29]. Survivin, therefore, promotes tumor proliferation directly or indirectly by regulating cancer cell homeostasis [30]. The results show that IWR-1 effectively decreased survivin expression in a dose- and time-dependent manner (Figure 4A–4B), suggesting possible effect of IWR-1 on the survivin.

We further investigated the effects of IWR-1 on EMT of HCT116 cells in which survivin expressions had been increased and decreased, respectively. Survivin overexpression was induced by transfecting HCT116 cells with pcDNA-survivin. The control group was transfected with empty pcDNA3.1 vector. Interestingly, IWR-1 could not effectively inhibit EMT in survivin-overexpressing HCT116 cells (Figure 4C). Next, survivin suppression was made by the treatment with si-RNA targeting survivin (si-Survivin). The control group was treated with scrambled siRNA (si-Control). EMT-suppressing effects of IWR-1 was more pronounced in survivin-suppressed HCT116 cells than in the control HCT116 cells (Figure 4D). Taken together, our results are consistent with the supposition that IWR-1 inhibits EMT by decreasing survivin expression.

Furthermore, we evaluated the effects of IWR-1 on MMP activities according to survivin expression status. In survivin-overexpressing cells (pcDNA-Survivin), IWR-1 could not effectively reduce gelatinase activities of MMP2 and MMP9 (Figure 4E). By contrast, IWR-1 could significantly reduce MMP activities when survivin was suppressed (si-Survivin) (*P* < 0.05) (Figure 4F). These findings are also consistent with the supposition that IWR-1 reduces MMP activities by decreasing survivin expression.

### Determination of DNA element within the survivin promoter where IWR-1 exerts its inhibitory effect of survivin transcription

**Figure 5:**
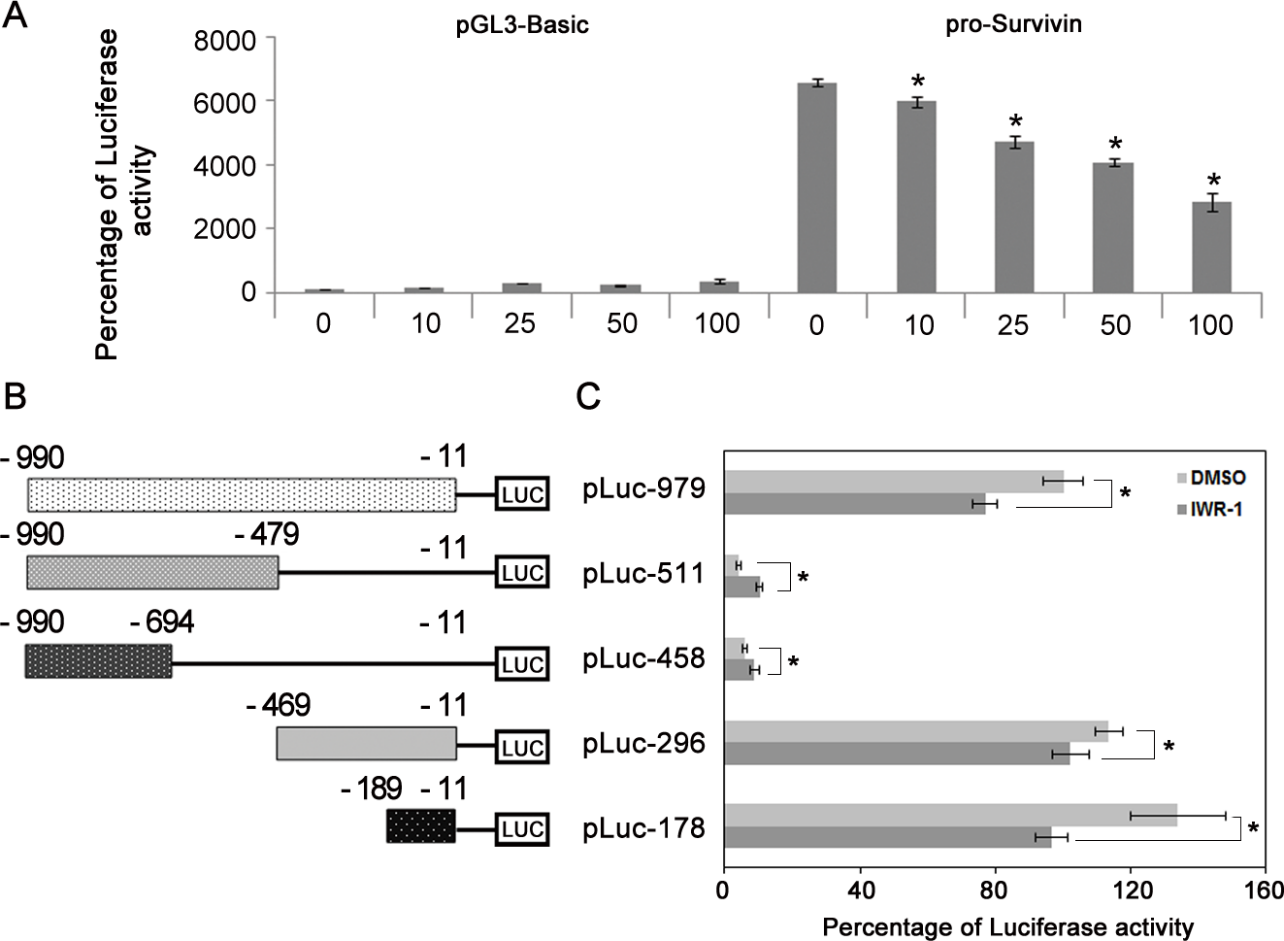
IWR-1 effects on survivin transcriptional activity and identification of DNA element responsible for inhibition of survivin transcription by IWR-1 **A.** Luciferase reporter assay demonstrating that the activity of the survivin promoter was dose-dependently decreased by IWR-1 treatment. The control was the pGL3-Basic-transfected cells. **B.** Schematic representation of the survivin promoter region. **C.** Identification of survivin promoter region responsible for inhibition of survivin transcription by IWR-1. HCT116 cells were transfected with various survivin promoter–luciferase constructs and treated with or without IWR-1 (10 μM) for 24 h after transfection, followed by luciferase activity assays. A 178-bp DNA element from −189 to −11 bp was identified playing a major role in IWR-1-mediated inhibition of survivin promoter activity. Data are shown in histograms and each bar is the mean ± SD derived from three independent assays in panels A and B. Luciferase activity was normalized to Renilla luciferase and expressed in arbitrary units.

Because we have shown that IWR-1 inhibits EMT by decreasing survivin expression, we conducted luciferase reporter assay to investigate whether IWR-1 could decrease survivin transcriptional activity. The control was the pGL3-Basic-transfected cells. The luciferase reporter assay demonstrated that the activity of the survivin promoter was dose-dependently decreased by IWR-1 treatment (Figure 5A).

We then attempted to identify survivin promoter region responsible for inhibition of survivin transcription by IWR-1 (Figure 5B, 5C). Series of survivin promoter-luciferase deletion constructs (−990/–479, −990/–694, −469/–11, and −189/–11) were generated from pLuc-979 using HindIII, HincII, SmaI, MluI, ApaLI restriction enzymes. HCT116 cells were then transfected with various survivin promoter–luciferase constructs and treated with or without IWR-1 (10 μM) for 24 h after transfection, followed by luciferase activity assays. A 979-bp survivin promoter region from −990 to −11 was identified as playing a major role in IWR-1-mediated inhibition of survivin promoter activity. Next, we narrowed the minimal responsive region in the survivin promoter using a set of contiguous deletion constructs derived from the 979-bp core promoter region. A 179-bp DNA element from −189 to −11 bp was identified playing a major role in IWR-1-mediated inhibition of survivin promoter activity.

### Demonstration of IWR-1 effects in CRC tissues

We then performed comparative western blotting analysis of paired surgical specimens (CRC tissues and non-cancerous colorectal tissues from the same patients) obtained from 13 patients that underwent treatment in our institution. The median patient age was 71 years (55–83). The median tumor size was 5.0 cm (1.5–12.0), and the lesions were distributed in the cecum (*n* = 1), descending colon (*n* = 1), sigmoid colon (*n* = 8), and rectum (*n* = 3). Histopathology of CRC tissues revealed moderately differentiated adenocarcinoma (*n* = 11) and poorly differentiated adenocarcinoma (*n* = 2). The patients showed stage I (*n* = 2), stage II (*n* = 3), and stage III (*n* = 8) CRCs.

**Figure 6:**
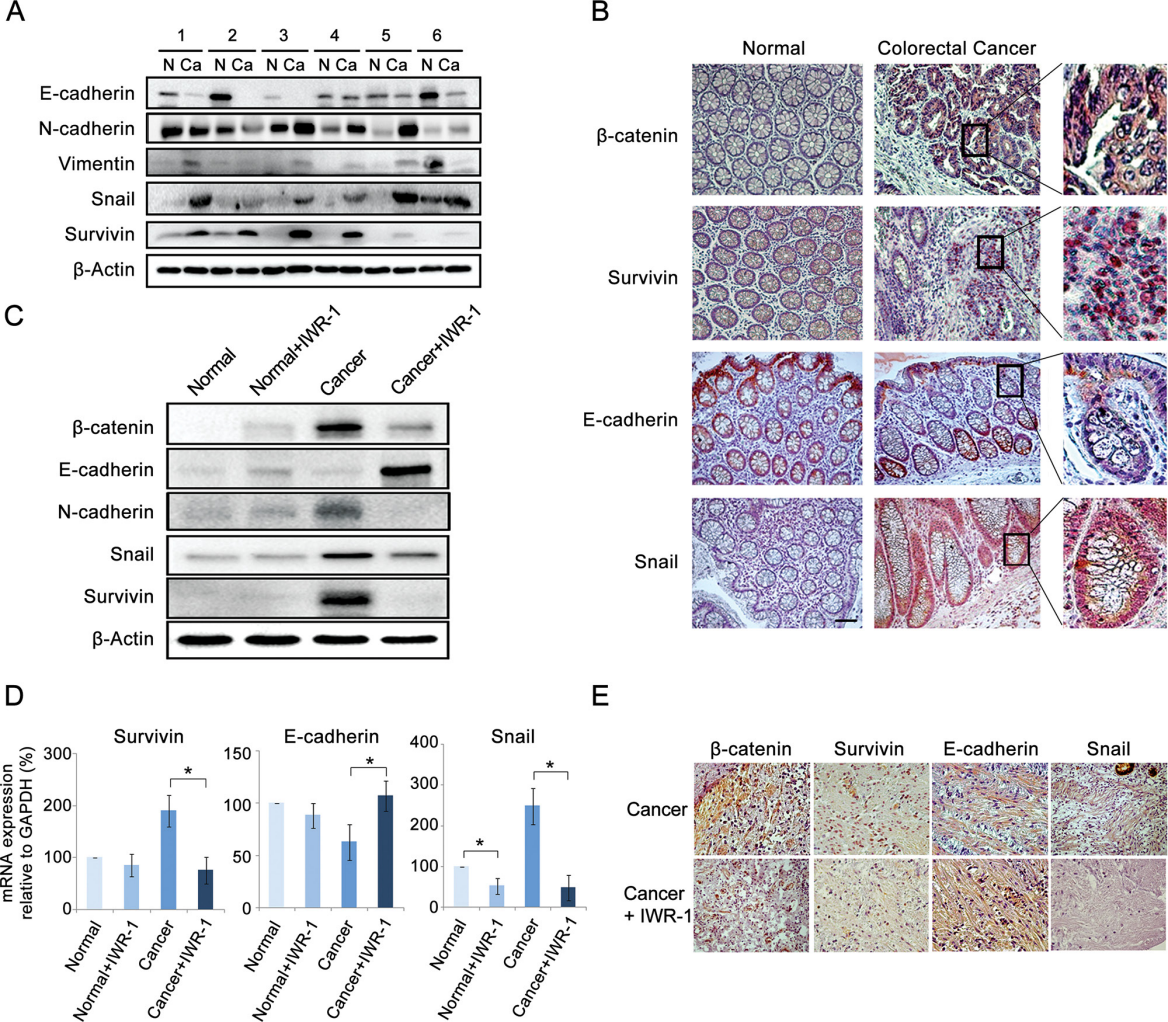
IWR-1 suppression of the EMT in the *ex vivo* model of colorectal cancer **A.** Western blot analysis showing that colorectal cancer (CRC) specimens exhibited the increased survivin expression and EMT-like expressional changes (decreased E-cadherin and increased N-cadherin and Snail expressions) compared to the matched controls. **B.** Immunohistochemical analysis of CRC tissues showing the increase of β-catenin (purple color), survivin (purple-to-red color), and Snail (red color) and decrease of E-cadherin (disappearing of red color) (Magnification, ×100, scale bar 50 μM). Generally, positive expressions of β-catenin, survivin, E-cadherin, and Snail were stained in purple-to-red colors. **C.** Western blot analysis showing IWR-1 effects on EMT markers in CRC tissues. IWR-1 significantly decreased the protein expressions of β-catenin and survivin, and inhibited EMT in CRC tissues. **D.** RT-qPCR showing IWR-1 effects on the mRNA expressions of the EMT markers in CRC tissues. IWR-1 significantly decreased the mRNA expressions of β-catenin and survivin, and inhibited EMT in CRC tissues. **E.** Immunohistochemical stains demonstrating that IWR-1 increased E-cadherin and decreased survivin and Snail expressions in CRC tissues.

Western blot analysis showed that CRC specimens exhibited the increased survivin expression and EMT-like expressional changes (decreased E-cadherin and increased N-cadherin and Snail expressions) compared to the matched controls (Figure 6A). Subsequently, immunohistochemical analysis showed the increase of β-catenin, survivin, and Snail and decrease of E-cadherin in CRC tissues (Figure 6B).

The effects of IWR-1 on the expression of EMT markers and survivin were further investigated in the *ex vivo* model. CRC tissues were cultured with or without 10 ng/ml TNF-α and/or 10 μM IWR-1 for 24 h, and were compared using western blot analysis. IWR-1 significantly inhibited EMT in an *ex vivo* model; it significantly decreased the protein expressions of β-catenin and survivin, and inhibited EMT of CRC tissues, even in the presence of TNF-α-induced EMT stimulation (Figure 6C). RT-qPCR and immunohistochemical stains also showed IWR-1 increased E-cadherin and decreased survivin and Snail at the mRNA and protein levels, respectively (Figure 6D, 6E).

## DISCUSSION

The present study demonstrates that IWR-1, an inhibitor of the Wnt/β-catenin pathway, has the potential to suppress tumor invasion and metastasis in both *in vitro* (HCT116 and HT29 cells) and *ex vivo* (CRC patient-derived tissues) CRC models: IWR-1 showed clear and consistent inhibition of CRC cell proliferation, invasion, migration, EMT process, and MMP activities. These potentials to suppress CRC invasion and metastasis were evident even after TNF-α-induced EMT stimulation. The anticancer effect of IWR-1 has been attributed to the enhancement of β-catenin destruction by way of blocking Axin protein turnover. In addition to this, we have found that IWR-1 reduces the expression of an anti-apoptotic protein survivin. Survivin promotes tumor proliferation by way of modulating multiple critical cell signaling pathways. Therefore, IWR-1 is expected to provide more powerful potential to suppress tumor invasion and metastasis, representing as a promising strategy for future CRC treatment.

In this study, we have shown that IWR-1 significantly inhibits PI3K/Akt signaling pathway by decreasing survivin expression. Akt, initially described as an oncogene [31, 32], serves a variety of functions, including cell survival, proliferation, cell cycle regulation, and EMT [33]. A number of studies have indicated that Akt activation in human cancer is a major determinant of aggressive clinical behavior [34–41]. We have found that IWR-1 inhibited Akt expression in a dose- and time-dependent manner. However, interestingly, when Akt was overexpressed by the transfection with pcDNA3.1Myr-Akt, IWR-1 still exerted the EMT reversal potential. Therefore, it can be postulated that IWR-1 acts on the downstream molecule of Akt signaling in favor of EMT rather than directly acting on the Akt itself. Survivin is a downstream molecule of Akt signaling in favor of EMT [42–44]. In this study, we showed the evidence that IWR-1 provides EMT reversal effects by directly suppressing survivin expression by the followings: 1) IWR-1 could not completely inhibit EMT in survivin-overexpressing HCT116 cells, 2) EMT reversal effects of IWR-1 were more pronounced in survivin-suppressed cells than in the controls, and 3) Survivin promoter assay directly identified the survivin promoter region responsible for inhibition of survivin transcription by IWR-1. Taken altogether, it could be concluded that IWR-1 inhibits EMT of CRC cells by way of decreasing survivin (its downstream substance) expression.

In this study, we also found that IWR-1 also consistently reduced MMP activities of CRC cell lines. The degradation of the ECM by proteolytic enzymes is a prerequisite to invasion and metastasis in EMT [45], and the principal ECM-degrading enzymes are MMP2 and MMP9. Metastatic cancer cells actively synthesize MMPs, the higher levels of which reflect enhanced tumor metastatic capacity [25, 46, 47]. We have shown that IWR-1 significantly reduces the MMP2 and MMP9 activities in the cancer cells, even in the presence of TNF-α-induced cancer cell stimulation. Furthermore, we have found that 1) IWR-1 does not completely reduce MMP activities in survivin-overexpressing HCT116 cells, and 2) IWR-1 reduces MMP activities especially when survivin expression is suppressed. Collectively, these findings suggest that IWR-1 reduces MMP activities by decreasing survivin expression.

Survivin is preferentially and highly expressed in cancer cells, and promotes tumor proliferation. Several mechanisms by which survivin promotes tumor progression have been proposed. First, survivin confers resistance to apoptosis by directly suppressing caspase-3 activity [48]. In this signaling pathway, survivin binds to and inhibits caspase-3 through the phosphorylation of Thr34 by the p34 (cdc2) kinase [49]. Second, survivin controls chromosome spindle-checkpoint assembly, thereby ensuring normal cell division [50]. It was evidenced by its maximal expression during G2/M checkpoint of the cell cycle [51–53]. Finally, survivin serves a critical function in tumor angiogenesis as it is strongly expressed in endothelial cells during the proliferative phase of angiogenesis [54–56]. Therefore, survivin is highly expressed in patients with an increased risk of tumor progression and chemoresistance in various tumors [57–59].

Besides, several reports indicate that survivin favors EMT based on the observation of increased survivin expression during EMT [13, 60–62]. However, it has not thoroughly investigated whether and how much survivin is associated with the EMT process of CRC cells. Herein, we clearly showed that survivin is increased during the EMT process in both *in vitro* and *ex vivo* CRC models. To determine the role of survivin in EMT with certainty, we designed HCT116 cells which have been manipulated to overexpress or suppress survivin, respectively. Whereas survivin-overexpressing HCT116 cells increased EMT, survivin-suppressed cells did not increase EMT. These results strongly suggest that overexpressed survivin has a detrimental effect on CRC prognosis by promoting EMT, and therefore, controlling survivin would be one of the possible strategies to prevent CRC progression.

**Figure 7:**
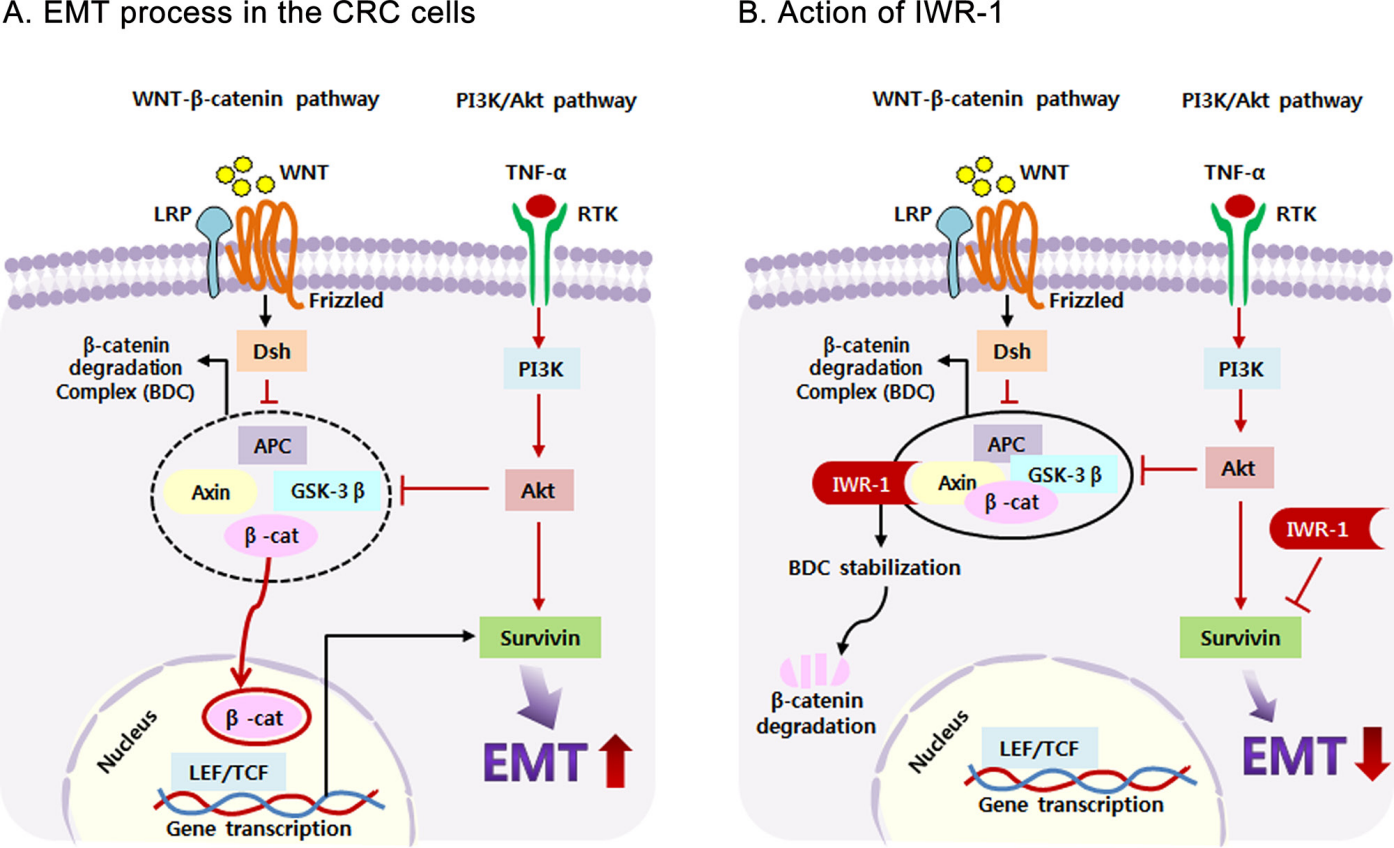
Proposed mechanism of IWR-1 related to EMT and survivin in colorectal cancer cells **A.** EMT process in the colorectal cancer (CRC) cells. **B.** Action of IWR-1. Activation of the Wnt/β-catenin and PI3K/Akt pathways promotes EMT by increasing survivin expression. IWR-1 is known to block Wnt/β-catenin signaling by promoting β-catenin destruction in the β-catenin destruction complex. In this study, we have shown that IWR-1 can also inhibit EMT progression of CRC cells by directly suppressing survivin expression.

Based on these results, we propose a mechanism by which IWR-1 elicits its EMT reversal effect of CRC cells (Figure 7A, 7B). We herein focused on the two pathways, Wnt/β-catenin and PI3K/Akt, of which activations have been shown to promote EMT. As reported, IWR-1 increases β-catenin destruction by way of blocking Axin protein turnover, thereby resulting in exerting its EMT reversal abilities. Wnt/β-catenin signaling is one of the key signaling pathways triggering EMT, and survivin is a known downstream target of Wnt/β-catenin signaling [21, 22]. Therefore, IWR-1, as a Wnt/β-catenin inhibitor, seems to decrease survivin in the process of inhibiting EMT. Of note, survivin is also one of the downstream substances of PI3K/Akt signaling [19, 63]. In this study, we have shown the evidence that IWR-1 inhibits EMT process by way of directly decreasing survivin expression. Because survivin is the downstream substance of both Wnt/β-catenin and PI3K/Akt signaling pathways targeting EMT, IWR-1 is expected to have higher EMT reversal abilities.

In summary, we have shown that survivin serves critical functions as a downstream substance of both Wnt/β-catenin and PI3K/Akt signaling pathways triggering EMT. IWR-1, as a Wnt/β-catenin inhibitor, exhibited strong potentials to suppress CRC cell invasion and metastasis, especially in terms of suppressing migration, invasion, EMT, and MMP activities of CRC cells. Besides inhibiting Wnt/β-catenin signaling pathway, IWR-1 was demonstrated to inhibit EMT by way of decreasing survivin expression. This work thus lays the foundation for a novel, IWR-1-based therapeutic approach to prevent CRC progression. Clinical studies are required to identify patient populations that will greatly benefit from IWR-1 therapy.

## METHODS

### Chemicals and reagents

IWR-1 was obtained from ENZO (Farmingdale, NY, USA), PI3K inhibitor LY294002 was purchased from Sigma-Aldrich (St Louis, MO, USA), and recombinant human tumor necrosis factor (TNF)-α was from R&D Systems (Minneapolis, MN, USA). Primary antibodies against E-cadherin, N-cadherin, Snail, Survivin, Vimentin, p-Akt (Ser473), t-Akt, β-catenin, and β-Actin as well as secondary antibodies conjugated to horseradish peroxidase (HRP), Alexa-594, and FITC were obtained from Cell Signaling Technology (Danvers, MA, USA). Lipofectamine 2000, SYBR Green, and the total RNA purification kit were purchased from Invitrogen (Carlsbad, CA, USA), and the RT-Premix Kit was obtained from ELPIS Biotech (Daejeon, South Korea).

### Cell culture

We obtained four colorectal carcinoma cells, HCT116, HT29, SW480, and SW620 from KCLB (Korean cell line bank). They were maintained in RPMI medium (Thermo Fisher Scientific, Carlsbad, CA, USA) supplemented with 10% fetal bovine serum (FBS) and 1% penicillin-streptomycin (GibcoBRL, Carlsbad, CA, USA) at 37°C in an atmosphere of 5% CO_2_ in a humidified incubator.

### Cell proliferation assay

Cell proliferation was evaluated by the water soluble tetrazolium (WST-1) assay using the EZ-Cytox Cell proliferation kit (Itsbio, Seoul, Korea) according to the manufacturer’s instruction. Briefly, HCT116 and HT29 cells were plated in 96-well plates at a density of 1 × 10^4^ cells/well overnight and then treated with different concentrations of IWR-1 (5, 10, 25, and 50 μM) for 24 h or 48 h, respectively. Next, the reagent from the EZ-Cytox kit was added to each well for 30 min; the plates were then placed on a shaker for 30 s and the absorbance was measured at 450 nm using a microplate reader (model 680; Bio-Rad, Hercules, CA, USA).

### Western blot analysis

Cells were lysed using the EzRIPA Lysis kit (ATTO Corporation; Tokyo, Japan), and quantified by Bradford reagent (Bio-Rad). Proteins were visualized by western analysis using the following primary antibodies (1:1000 dilution) at 4°C overnight and then with HRP-conjugated secondary antibodies (1:2000 dilution) for 1 h at 25°C. Specific immune complexes were detected using the Western Blotting Plus Chemiluminescence Reagent (Millipore; Bedford, MA).

### Real-time quantitative PCR

Total cellular RNA was extracted using TRIzol reagent (Invitrogen) according to the manufacturer’s instructions. Reverse transcription was performed with 1 μg RNA using RT-Premix kit (ELPIS Biotech, Daejeon, South Korea) according to the manufacturer’s instructions. The primers used for SYBR Green real-time quantitative (q) PCR were as follows: E-cadherin forward 5′-TACACTGCCCAGGAGCCAGA-3′ and reverse 5′-GGCACCAGTGTCCGGATTA-3′; Snail, forward 5′- GACCACTATGCCGCGCTCTT-3′ and reverse 5′-TCGCTGTAGTTAGGCTTCCGATT-3′; GAPDH, forward 5′- GCACCGTCAAGGCTGAGAAC-3′ and reverse 5′-TGGTGAAGACGCCAGTGGA-3′ [24]. The reaction was performed using an Applied Biosystems^®^ 7500 96-Well Real-Time PCR system (Life Technologies, Carlsbad, CA, USA). After normalization to the GAPDH gene, the expression levels for each target gene were calculated using the comparative threshold cycle method. The data are presented as the mean ± standard deviation (SD) from three independent experiments.

### Migration and invasion assays

HCT116 and HT29 cell migration was analyzed using the *in vitro* wound healing assay. Cells were grown to confluence in 48-well plates and changed to serum-free medium for additional 24 h. Cell monolayers were scraped with a micropipette tip and treated with 10 uM IWR-1. The wound area was photographed under phase-contrast microscopy before and 24 h after the treatment and the percentage of wound closure was determined as: [(initial area - final area)/initial area] × 100 [64].

Invasion assays were conducted using the CytoSelect 24-Well Cell Invasion Assay Kit (Cell Biolabs, San Diego, CA, USA). Briefly, 300 μL of HCT116 and HT29 cells (1 × 10^5^cells/ml) in serum-free medium was plated into the CytoSelect basement membrane chamber, respectively, and 500 μL of 10% FBS-containing RPMI was added to the lower well of the invasion plate; both upper and lower chambers contained the 10 uM IWR-1. The chambers were then incubated for 48 h at 37°C in a 5% CO_2_ atmosphere, non-migratory cells were removed, and migrated cells were stained, dissociated from the membrane, and their absorbance was measured at 560 nm using the microplate reader (model 680; Bio-Rad, Hercules, CA, USA) [65].

### Immunofluorescence

HCT116 and HT29 cells were cultured on Lab-Tek chamber slides (Thermo Fisher Scientific). The cells were washed three times with PBS, fixed with 4% paraformaldehyde for 20 min, and permeabilized with 0.3% Triton X-100 for 10 min. After blocking with 0.2% bovine serum albumin for 1 h at room temperature, the slides were incubated with the antibodies against E-cadherin, Vimentin, or Snail (1:100 dilution) at 4°C overnight. The slides were washed three times with PBS and incubated with Alexa Fluor 488- or Alexa Fluor 594-conjugated secondary antibodies (1:200 dilution) for 1 h at room temperature; the nuclei were counter-stained with DAPI-containing VECTASHIELD Mounting Medium (Vector Labs, Burlingame, CA, USA) for 1 min. The samples were then examined under a Laser Scanning Microscopy (Nikon, Tokyo, Japan) to analyze the expression of E-cadherin, Vimentin, and Snail.

### Gelatin zymography analysis of secreted matrix metalloproteinases

Cell culture supernatants were collected, centrifuged at 400 × *g* for 5 min, and mixed with 3× sample buffer (62 mM Tris-HCL, pH 6.8; 2% SDS; 10% glycerol; and 0.05% bromophenol blue). Zymography was performed using 10% polyacrylamide gels impregnated with 0.2% gelatin. Following electrophoresis, the gels were washed twice at room temperature for 30 min in 2.5% Triton X-100, then in buffer containing 50 mM Tris-HCl, 150 mM NaCl, 5 mM CaCl_2_, and 0.02% NaN_3_, pH 7.5, and incubated in this buffer at 37°C for 24 h. Then, the gels were stained with 0.5% (w/v) Coomassie Brilliant Blue G250 (Bio-Rad) for 1 h and destained in methanol-acetic acid-water (5:1:5 molar ratio). Gelatinolytic activity was identified by clear bands appearing on the blue-stained background. The gels were scanned, and the images were processed by extracting the blue channel signal, converting it to black and white, and inverting it to quantify gelatinolytic activity based on integrated optical density.

### Overexpression and silencing of the Akt and survivin genes

Human survivin cDNA and Akt cDNA were obtained by RT-PCR using HCT116 and HT29 cell RNA and primers based on GenBank sequences (accession numbers U75285 [48] and NM-001014431.1, [66] respectively). PCR products were digested with *Eco*RI and *Xho*I (Takara Tokyo, Japan), and ligated into the pcDNA3.1-myc vector (Invitrogen). Transcription was specifically suppressed by the introduction of the 21-nucleotide duplex siRNA, which targeted survivin mRNA coding sequence. Briefly, HCT116 cells were plated in 6-well plates (2 × 10^5^ cells/well) and transiently transfected with 100 nM per well of survivin siRNA (Santa Cruz) mixed with the DharmaFECT siRNA transfection reagent (Thermo Fisher Scientific) according to the manufacturer’s instructions. Silencer Negative Control siRNA (Santa Cruz) was used as a negative control and introduced into the cells under the same protocol. After 5-h incubation, the medium was changed to complete culture medium, and the cells were incubated at 37°C in a CO_2_ incubator for 48 h before harvesting.

### Collection and culture of colorectal cancer tissue

Human colorectal tissue specimens (paired normal and cancer tissues from each patient, *n* = 13) were obtained after surgical resections performed at the Daejeon St. Mary’s hospital, the Catholic University of Korea. The ethics committee at our institution approved the use of the tissue specimens for research. The tissue samples (diameter, 3 – 4 mm) were washed three times, dissected in cold PBS (Invitrogen) containing 2× penicillin/streptomycin mixture (Thermo Fisher Scientific), and cultured in 24-well culture plates with serum-free DMEM/F12 containing 2× penicillin/streptomycin and 10 μM IWR-1 to establish an *ex vivo* human cancer organ model. The plates were then placed in a humidified incubator and maintained for 24 h in an atmosphere of 5% CO_2_ at 37°C. Thereafter, the tissue samples were collected for western blot and fixed in 10% buffered formalin solution and embedded in paraffin for histology, respectively.

### Construction of the plasmid containing survivin promoter

Genomic DNA was extracted from HCT116 cells using the DNA extraction kit (Qiagen, Valencia, CA, USA). Survivin promoter site (979 bp) in the 5′ flanking region of the survivin gene was amplified by PCR using the following primers: forward primer, 5′-CTGGCCATAGAACCAGAGAAGTGA-3′ and reverse primer, 5′-CCACCTCTGCCAACGGGTCCCGCG-3′. Subsequently, the promoter region was excised using SacI and HindIII, and subcloned into the pGL3-Basic vector (Promega) cut with the above enzymes to generate the pGL3-survivin plasmid. Thereafter, to identify survivin promoter region responsible for inhibition of survivin transcription by IWR-1, the survivin promoter–luciferase deletion constructs (pLuc-511, pLuc-458, pLuc-296, and pLuc-178) were generated from pLuc-979 using HindIII, HincII, SmaI, MluI, ApaLI of restriction enzymes.

### Luciferase reporter assay

HCT116 cells (2 × 10^5^ per well of a 6-well plate) were transiently transfected with 1 μg of the pGL3-survivin-luciferase construct and pSV-β-galactosidase control vector (Promega) using Lipofectamine. After transfection for 24 h, HCT116 cells were treated with 10 μM of IWR-1 for 24 h, and then harvested using the lysis buffer. Luciferase activity in cell extracts was measured using the Dual-luciferase assay kit (Promega) and a luminometer (Berthold technologies, Bad wildbad, Germany); it was normalized for transfection efficiency using the β-galactosidase assay (Promega).

### Immunohistochemical analysis

Paraffin-embedded tissue sections were deparaffinized in xylene and rehydrated in a graded series of ethanol. The antigen was retrieved with 0.01 M citrate buffer (pH 6.0) by heating the sample in an autoclave (CHS-ACCE-860, JW Pharmaceutical, Seoul, Korea) at a controlled final temperature of 121°C for 5 min. The tissue sections were then placed in 3% hydrogen peroxide for 5 min to inactivate the endogenous peroxidase, blocked for 10 min with normal horse serum (DakoCytomation LSAB2 System- HRP kit; DakoCytomation, Glostrup, Denmark), and incubated with the primary antibodies against β-catenin, survivin, E-cadherin, and Snail overnight at 4°C. The slides were then treated with the biotinylated secondary antibody for 30 min at room temperature, followed by streptavidin-HRP and 3, 3′-diaminobenzidine solution for another 10 min at room temperature.

### Statistical analysis

All data were analyzed using the SPSS 11.0 software (SPSS Inc.; Chicago, IL, USA) and are presented as the mean ± SD. Statistical comparison between the mean values of two groups was performed using Mann-Whitney *U*-test; to compare three or more groups, the Kruskal-Wallis test was used. Probability (*P*) values of < 0.05 were considered statistically significant.

## SUPPLEMENTARY FIGURES


