# Supplementary material for: IWR-1 inhibits epithelial-mesenchymal transition of colorectal cancer cells through suppressing Wnt/β-catenin signaling as well as survivin expression
Source: Oncotarget. 2015 Sep 16;6(29):27146–59. doi: 10.18632/oncotarget.4354 (PMC4694979; doi:10.18632/oncotarget.4354)
Supplement: Supplementary file 1 [file oncotarget-06-27146-s001.pdf]

SUPPLEMENTARY FIGURES

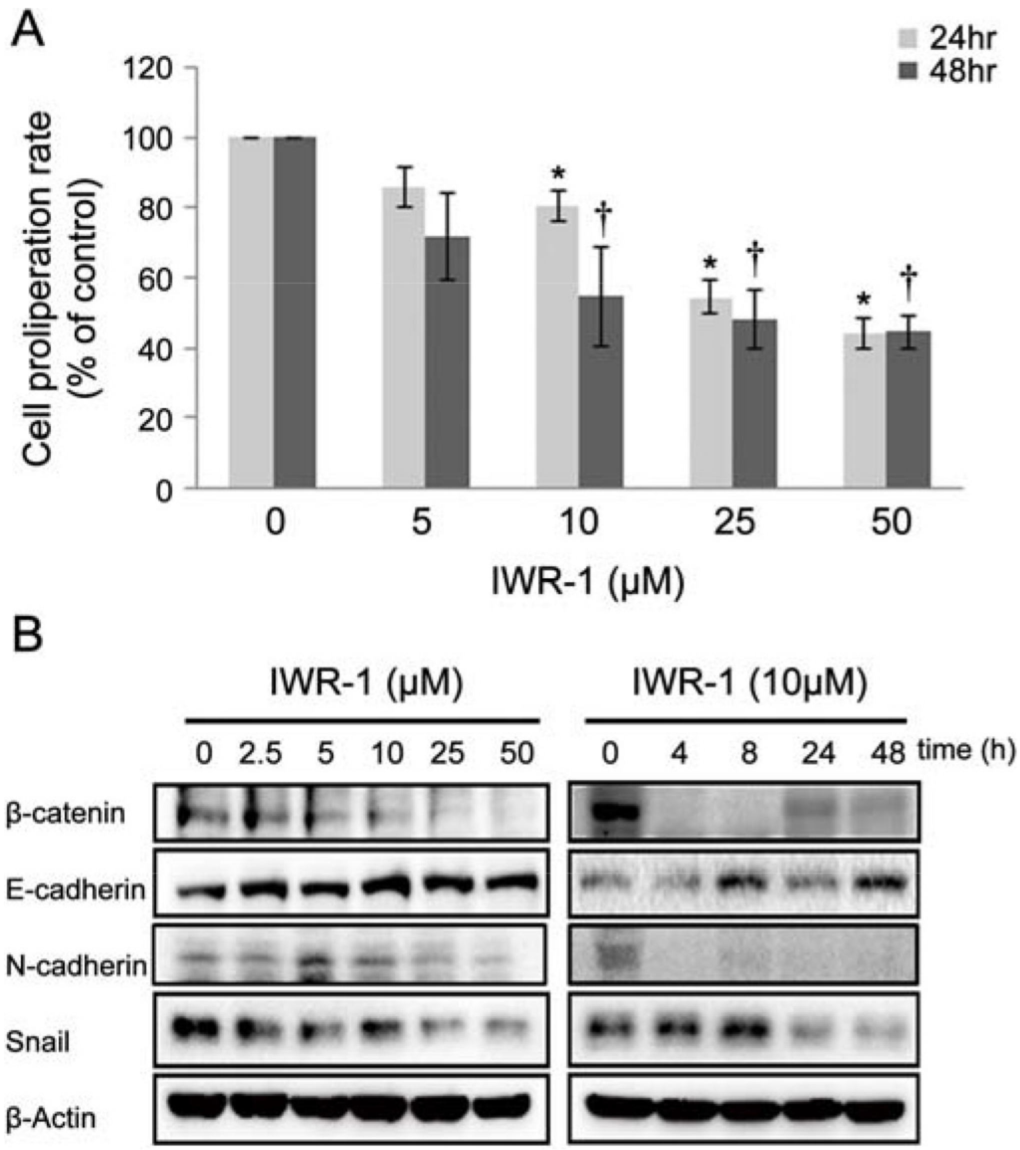

Supplementary Figure S1: IWR-1 effects on HT29 cell proliferation and EMT. A. Treatment of HT29 cells with increasing concentrations of 10 μM IWR-1 for 24 h and 48 h. IWR-1 decreased the proliferation of HT29 cells in a dose- and time-dependent manner. **B.** Western blot analysis showing protein expression patterns of the EMT markers according to IWR-1 dose and exposure time. β-Actin was used as a loading control. Values represent means ± SD of three independent experiments. *$P < 0.05$, †$P < 0.05$.

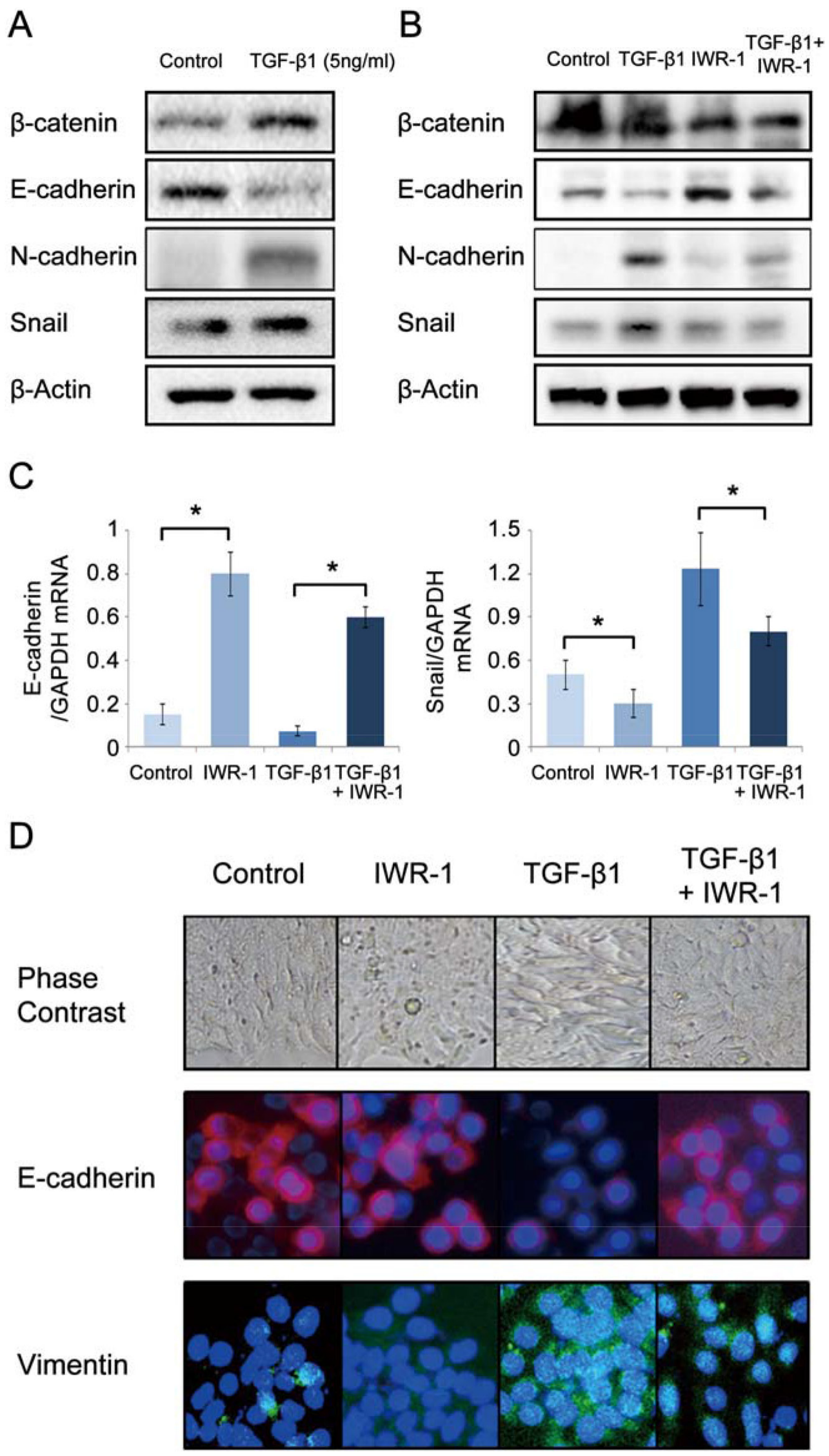

Supplementary Figure S2: IWR-1 effects on TGF-β1-induced EMT in HT29 cells. A. Western blot analysis showing that TNF-α increased the expression of β-catenin, induced EMT-like expressional changes, and caused a switch from E-cadherin to N-cadherin expression in HT29 cells. β-Actin was used as loading controls. **B.** Western blot analysis showing IWR-1 effects on the expressions of β-catenin and EMT markers. IWR-1 decreased the expression of β-catenin and inhibited EMT progression, even in the presence of TNF-α stimulation in HT29 cells. **C.** RT-qPCR showing that the mRNA levels of E-cadherin and Snail were increased and decreased after IWR-1 treatment, respectively ($P < 0.05$). **D.** Immunofluorescence analysis demonstrating the increase in E-cadherin and decrease in Vimentin after the treatment of IWR-1, even in the TGF-β1-stimulated HT29 cells. GAPDH was used as loading controls. Values represent the means ± SD of three independent experiments. $^*P < 0.05$

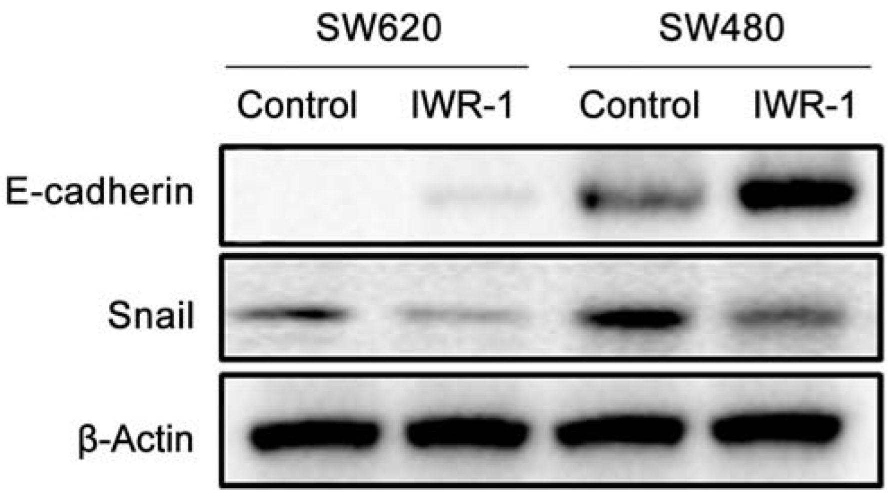

Supplementary Figure S3: IWR-1 effects on EMT of APC-mutated CRC cells. SW480 and SW620 cells were known APC-mutated colorectal cell lines. These cell lines were cultured in RPMI with or without IWR-1(10 μM) for 24 h and thereafter, EMT process was measured using western blot analysis. IWR-1 clearly inhibited EMT process of these APC-mutated colon carcinoma cell lines, demonstrated by increased epithelial marker E-cadherin and decreased mesenchymal marker Snail. β-Actin was used as a loading control. Values represent means ± SD of three independent experiments.

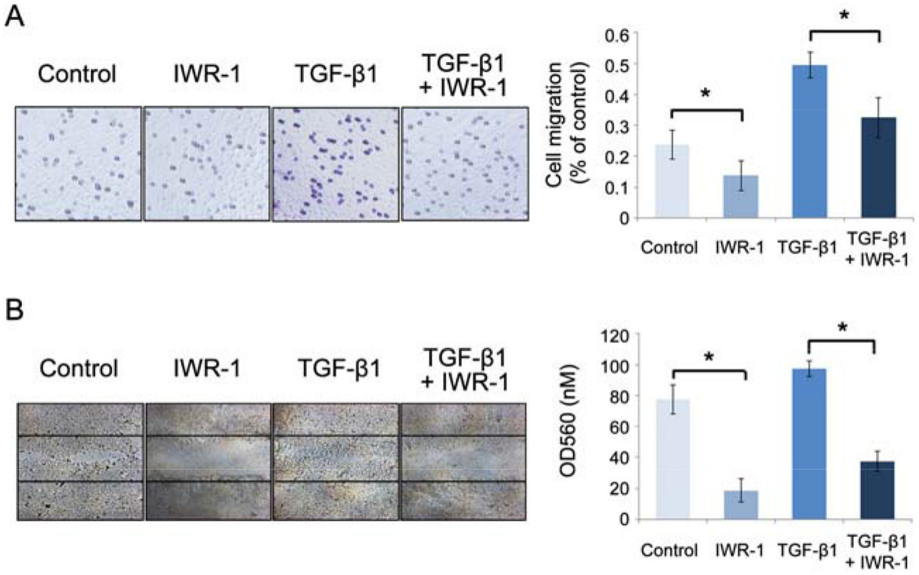

Supplementary Figure S4: IWR-1 effect on the TGF-β1-induced invasion and migration of HT29 cells. A. Transwell invasion assay (magnification, ×100, scale bar 20 μM) showing that IWR-1 significantly inhibited TNF-α-stimulated HT29 cell invasion ($P < 0.05$). **B.** Wound-healing assay (magnification, ×200, scale bar 50 μM) showing that IWR-1 significantly inhibited TNF-α-stimulated HT29 cell migration ($P < 0.05$).
